# Physicochemical Aspects of Oxidative Consolidation Behavior of Manganese Ore Powders with Various Mn/Fe Mass Ratios for Pellet Preparation

**DOI:** 10.3390/ma15051722

**Published:** 2022-02-25

**Authors:** Yuanbo Zhang, Bei Zhang, Bingbing Liu, Junjie Huang, Jing Ye, Yuelong Li

**Affiliations:** 1 School of Minerals Processing and Bioengineering, Central South University, Changsha 410083, China; sintering@csu.edu.cn; 2 School of Chemical Engineering, Zhengzhou University, Zhengzhou 450001, China; abyzzu@126.com; 3 Baosteel Resources Holding (Shanghai) Co., Ltd., Shanghai 200080, China; huangjunjie1102@baosteel.com (J.H.); ye_jing@baosteel.com (J.Y.); liyuelong@baosteel.com (Y.L.)

**Keywords:** manganese ore pellet, Mn/Fe mass ratio, microstructure, phase transformation, consolidation

## Abstract

With the depletion of rich manganese ore resources, plentiful manganese ore powders with various Mn/Fe mass ratios are produced. The physicochemical aspects of oxidative consolidation behavior of manganese ores with various Mn/Fe mass ratios were investigated in this work to determine whether manganese ore powders with high iron content (Fe-Mn ore) can be prepared as high-quality pellets. Physicochemical properties of the pellets were investigated, including cold compression strength (CCS), phase transformation, microstructural evolution, Vickers hardness (HV), porosity, and lattice parameter. CCS testing indicated that the strength of roasted Fe-Mn ore pellets was observably lower than that of pure hematite or manganese ore pellets. Phase and morphology results showed that in Fe-Mn ore pellets, an Mn ferrite phase was generated between hematite and pyrolusite particles. However, newborn Mn ferrites and hematite had an obvious crystal boundary in the crystallographic particles. Moreover, poorly crystallized Mn ferrite particles were evident, along with Mn and Fe element concentration gradients, due to the inadequate diffusion of metal ions. This resulted in poor mechanical properties of the Fe-Mn ore pellets. A temperature over 1275 °C and a roasting time of 15 min is required for the oxidative consolidation of Fe-Mn ores. In such optimized cases, Mn, Fe, O, and Al elements were uniformly distributed in the well-crystallized Mn ferrite grains, which provided favorable mineralogy for the consolidation of Fe-Mn ore powders.

## 1. Introduction

Manganese and iron are strategic metal elements, and 90–95% of manganese, in the form of Mn alloys, is consumed in steel production [1]. With the depletion of rich manganese ore resources, low-grade manganese ores are becoming alternative resources for the extraction of manganese [2,3,4]. As reported, almost half of manganese ore resources in India are ferruginous, and more than 73% of manganese ores in China are ferruginous abbr. Fe-Mn ore), with a low Mn/Fe mass ratio (<3). During the exploitation and beneficiation of low-grade manganese ores, manganese ore powders with various Mn/Fe mass ratios are produced and need immediate attention [5,6,7]. Utilization of the abundant Fe-Mn ore powders for the production of Mn alloys is significant for the sustainable development of iron and steel manufacturing.

According to industrial classifications of manganese ore types by Mn/Fe mass ratio, manganese ores are divided into rich manganese ore (TMn > 30%, Mn/Fe mass ratio > 3), Fe-Mn ore (15% < TMn < 25%, Mn/Fe mass ratio < 3), and poor manganese ore (TMn < 15%) [5,8]. Manganese ore powders with various Mn/Fe mass ratios can refer to these three types of manganese ores. Pyrometallurgical smelting of manganese ores in blast furnaces and electric furnaces constitutes the mainstream production process for Mn alloys in factories [9,10,11,12,13]. In these processes, manganese ore fines or powders are first agglomerated to sinters or pellets, which have superior mechanical and metallurgical properties. Then, the agglomerates, as one kind of burden, are charged into the blast furnace or electric furnace for reduction smelting [14]. Therefore, it is of practical significance to investigate the oxidative consolidation of manganese ore with various Mn/Fe mass ratios to supply qualified furnace burdens.

Previous research has mainly concentrated on the characterization, as well as pelletizing and sintering behaviors, of pure iron ores and manganese ores with very high Mn/Fe mass ratios [15,16,17,18,19]. Faria et al. investigated the high-temperature disintegration of a Brazilian rich manganese lump ore and roasted pellets and found that the pellets demonstrated better mechanical and metallurgical properties than the natural lump ore [17,18]. The pelletization of rich manganese ore powders containing high combined water was investigated via raw-material pretreatment by high-pressure roll grinding to obtain pellets with high mechanical strength [20]. Zhao found that the main minerals in high-basicity sinters from manganese carbonate ore fines were calcium manganate, manganosite (MnO), and Mn-bearing silicate [21]. Lin reported that dominating minerals in the acid sinters of rich manganese oxide ore fines were hausmannite (Mn_3_O_4_), manganosite (MnO), and manganous silicate (2MnO·SiO_2_, MnO·SiO_2_) [22]. Sintering of ore fines and pelletization of ore powders are the two main processes for the preparation of qualified burdens for blast-furnace or electric-furnace smelting. In our previous study, we investigated the optimization of sintering parameters and consolidation behavior of Fe-Mn ore fines with an Mn/Fe mass ratio of 0.74 and natural basicity [13,23]. The tumbling index and yield of the Fe-Mn ore sinters were observably lower than those of pure iron ore sinters. As a result, the dosage of coke breeze required for the Fe-Mn ore (9.6–9.9 wt.%) was higher than that required for iron ores (4–5 wt.%) [22,23,24,25]. This suggests that the Mn/Fe mass ratio has a remarkable impact on the consolidation of manganese ores. The current work aims to prepare pellets from manganese ore concentrates with various Mn/Fe mass ratios.

In terms of mineralogy, the primary minerals in low-grade manganese are pyrolusite, hematite, and quartz. In order to systematically study the effect of various Mn/Fe mass ratios on the consolidation behavior of manganese ores, pure hematite ore mixed with a wide range of Mn/Fe mass ratios (0–12.25) was investigated. In the current study, bench-scale pelletizing and roasting experiments were conducted to determine the cold compression strength (CCS), phase transformation, morphology evolution, and element distribution of the roasted pellets via X-ray diffraction (XRD), Rietveld refinement, optical microscopy, scanning electron microscopy—energy-dispersive X-ray spectroscopy (SEM-EDS) line scanning, and HV hardness analyses. The reaction interface between hematite and manganese oxide particles was also characterized. Eventually, based on the findings, some suggestions were recommended for the preparation of satisfactory Fe-Mn ore pellets.

## 2. Experimental

### 2.1. Materials

**Figure 1:**
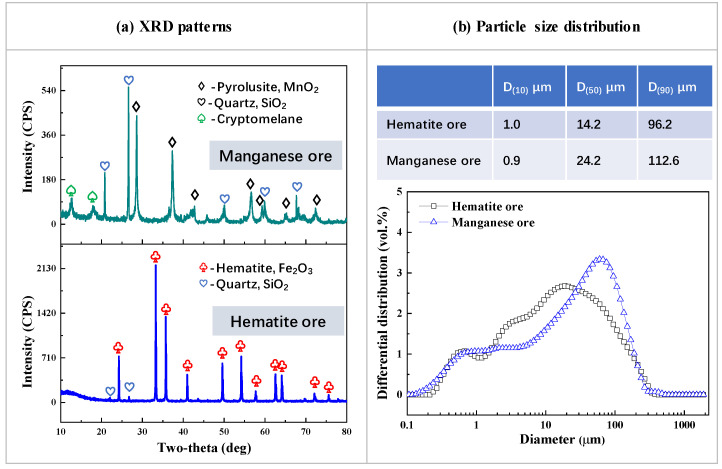
XRD patterns (**a**) and particle size distribution (**b**) of hematite ore and manganese ore.

Pure hematite ore, manganese oxide ore powders, and mixtures thereof were used as the raw materials. XRD patterns of the ores are displayed in Figure 1a. The main phases of hematite ore are hematite (Fe_2_O_3_) and quartz (SiO_2_), and the main mineral compositions in manganese ore are pyrolusite (MnO_2_), cryptomelane (Mn_8_O_16_·H_2_O), and quartz (SiO_2_). The total manganese and iron concentration of manganese ore and hematite is 47.80 wt.% and 64.76 wt.%, respectively, and the Mn/Fe mass ratio of manganese ore and hematite is 0 and 12.25, respectively. The detailed chemical compositions of the ore materials are listed in Table 1. Particle size distributions of hematite and manganese ore powders were measured with a Malvern laser particle-size analyzer (Mastersizer 2000, Malvern Panalytical, Malvern, UK) and are shown in Figure 1b. The average grain sizes of the hematite and manganese ore powders are 14.2 μm and 24.2 μm, respectively.

### 2.2. Methods

The experimental procedure comprised pellet preparation, oxidative preheating and roasting tests, CCS and porosity measurement, mineralogical and micro-area elemental distribution analyses, HV hardness measurements, and determination of lattice parameters.

To begin, the manganese ore and hematite powders were batched and mixed with various Mn/Fe mass ratios: 0 (sample H0), 0.05 (H1), 0.1 (H2), 0.15 (H3), 0.2 (H4), 0.25 (H5), 0.3 (H6), 0.4 (H7), 0.6 (H8), 0.8 (H9), 1.0 (H10), 1.2 (H11), 1.5 (H12), 1.8 (H13), 2.0 (H14), 2.5 (H15), 3.0 (H16), 3.5 (H17), 5.0 (H18), and 12.25 (H19). The batching scheme and chemical compositions of the hematite and manganese ore mixtures are listed in Table 1. Sample H0 refers to the pure hematite ore (Fe ore), and H19 represents the pure manganese ore (Mn ore). Fe-Mn ore is defined as low-grade manganese ore with an Mn/Fe mass ratio of less than 3, and thus, samples H1-H16 refer to Fe-Mn ores. The mixture was then combined with 1.5 wt.% bentonite binder with 8 wt.% moisture and then balled in a disc pelletizer with a diameter of 500 mm. The green balls with a diameter of 12–16 mm were statically dried at 105 °C for 4 h in a drying oven for subsequent preheating and roasting.

#### 2.2.1. Oxidative Roasting Procedure for Pellet Preparation

**Figure 2:**
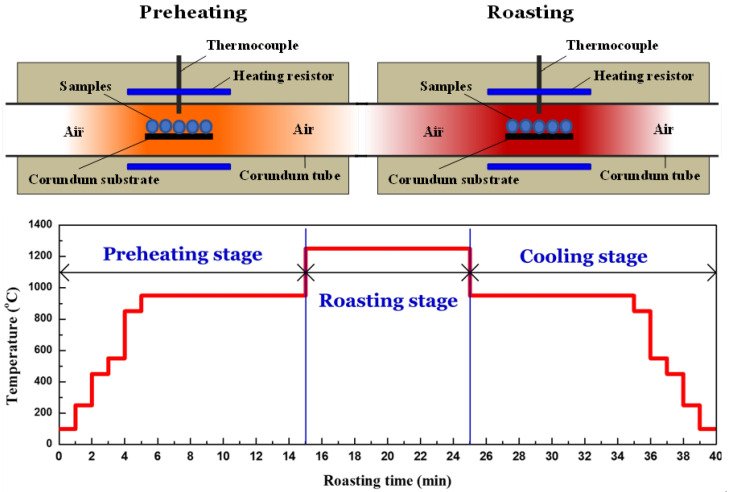
Schematic diagram of the oxidization roasting equipment and temperature curve.

In order to simulate the preheating and roasting process of pellets in the industrial grate-kiln process, an oxidation roasting experiment was conducted in a horizontal resistance furnace with two roasting areas. A schematic diagram of the oxidization roasting equipment and temperature curve are displayed in Figure 2. The dried pellets were placed on a corundum substrate (size: 80 mm in length and 10 mm in width) and loaded into the corundum tube with a diameter of 50 mm and a length of 1200 mm. Both ends of the corundum tube were open to the ambient atmosphere. In each batch experiment, the pellets were first moved from the left to the right and then further moved back to the left of the furnace, according to the designed temperature curves. The left part of the furnace was used for preheating and cooling, and the right part was used for roasting of the pellets. The temperature and time intervals of the preheating and cooling stages were fixed at 950 °C and 10 min, respectively. After roasting at given temperatures (1100–1300 °C) for a certain duration, the pellets were pulled out of the furnace and cooled to room temperature in the air atmosphere for subsequent characterization measurements. Notably, when the pellets were roasted at a certain temperature for 1 min during the simulative preheating and cooling process, the pellets were then instantly pushed to the next area for further roasting or cooling. Therefore, direct increases and drops in temperature are observed in Figure 2. Notably, temperature and interval were fixed as 950 °C and 10 min, respectively, for all preheating and cooling processes.

#### 2.2.2. Characterization

The cold compression strength (CCS) of the pellets was measured by a compression tester. An average value of 20 pellets was calculated and used as the final CCS for each test, and the standard deviation of all effective CCS results was 5%. CCS tests of the roasted pellets were conducted according to the standard of ISO 4700, 2015, Iron ore pellets for blast furnace and direct reduction feedstocks—Determination of the crushing strength [26]. The porosity of the roasted pellets was measured by the drainage method, according to GB/T 24586–2009, Iron ore or pellet- Determination of apparent density, true density, and porosity [27].

X-ray diffraction (XRD) patterns of the finely ground raw hematite, manganese ores, and finished pellets were detected by a diffractometer (Ultima IV, Rigaku Corporation, Tokyo, Japan) under the following radiation conditions: Cu Kα, tube current, and voltage: 40 mA, 40 kV; scanning range: 10–80° (2θ); step size: 0.02° (2θ); scanning speed: 8°/min. XRD-Rietveld refinement by TOPAS software was used to analyze the lattice parameters of the main ferrite phase [28], and in this case, the scanning speed was 2°/min.

After oxidization roasting, some representative finished pellets were mounted in resin and polished to a sections with a mirror surface. Then, the polished sections were examined by an optical microscope (MDI5000 M, LEICA, Wetzlar, Germany) and a scanning electron microscope (SEM, Helios G4 CX, Thermo Scientific, Waltham, MA, USA) equipped with an energy-dispersive X-ray spectroscopy (EDS) detector for microregion element analysis.

The evolution of microhardness for the manganese ferrite was investigated by a Vickers-type hardness tester (Model: HMV-2T, Shimadzu, Japan), using an applied load of 200 g and a dwell time of 15 s. The microhardness measurements for each tested specimen were repeated eight times in order to obtain precise results.

## 3. Results and Discussion

### 3.1. CCS Testing

**Figure 3:**
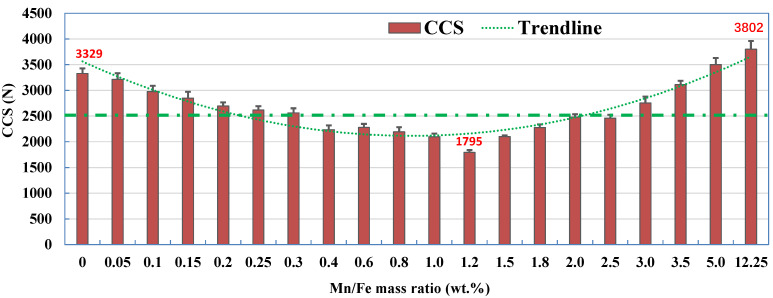
CCS of pellets with various Mn/Fe mass ratios after roasting at 1250 °C for 10 min.

Figure 3 shows the effect of the Mn/Fe mass ratio on the CCS of pellets with various Mn/Fe mass ratios (0~12.25) after roasting at 1250 °C for 10 min. In general, the CCS of pellets first decreases and then increases with the increasing Mn/Fe mass ratio from 0 to 12.25 but with a nadir at the Mn/Fe mass ratio of 1.2. The CCS of the pure hematite ore (Mn/Fe mass ratio of 0) and the manganese ore (Mn/Fe mass ratio of 12.25) is 3329 N and 3802 N, respectively. The CCS of the manganese ore pellets combined with hematite ore is lower than that of the pure manganese ores. The CCS of the pellets with an Mn/Fe mass ratio of 1.2 is only 1795 N. In particular, the Fe-Mn ore pellets with an Mn/Fe mass ratio in the range of 0.4–2.0 have a CCS of less than 2500 N, indicating these pellets cannot satisfy the strength criteria of pellets required for a large blast furnace.

### 3.2. Phase Transformation

**Figure 4:**
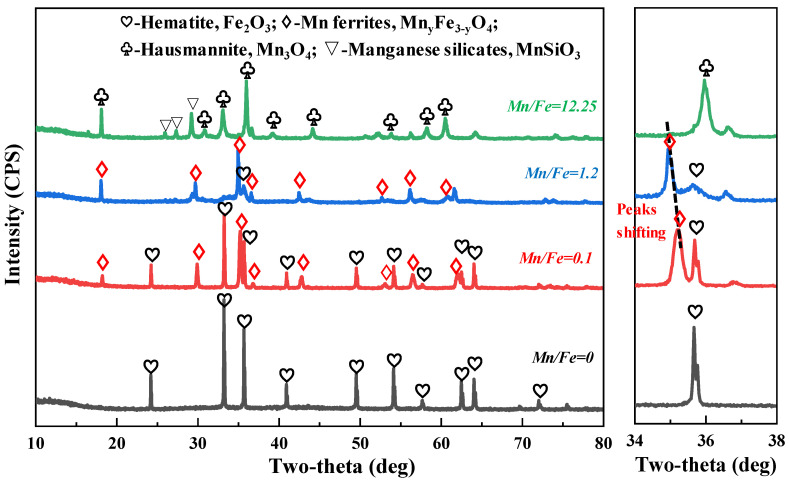
XRD patterns of pellets with various Mn/Fe mass ratios after roasting.

Figure 4 illustrates the XRD patterns of the pellets with Mn/Fe mass ratios of 0 (H0), 0.1(H2), 1.2 (H14), and 12.25 (H19) after oxidization roasting at 1250 °C for 10 min. As for the pure hematite ore with an Mn/Fe mass ratio of 0, the main phase is hematite (Fe_2_O_3_). When manganese ore is added to the hematite ores, diffraction peaks of Mn ferrites (Mn_y_Fe_3−y_O_4_) appear, and the intensity of the Mn ferrite peaks is enhanced, while those of hematite decrease. This indicates that the main phase of the Fe-Mn ores comprises Mn ferrites and hematite. With regard to the pure manganese ore with an ultrahigh Mn/Fe mass ratio of 12.25, the main consolidation phases are hausmannite (Mn_3_O_4_) and manganese silicates (MnSiO_3_). Moreover, the characteristic diffraction peak with an intensity of 100% (311, main peak) gradually shifts from 2θ = 35.19° towards 2θ = 34.93° with the increase in Mn/Fe mass ratio from 0.1 to 1.2, and the intensity of the diffraction peaks of Mn_y_Fe_3−y_O_4_ increase with increasing Mn/Fe mass ratio. Compared with the consolidation phase of pure hematite ore and manganese ore, newly generated Mn_y_Fe_3-y_O_4_ is the primary phase of the Fe-Mn ores. This is ascribed to the reaction between hematite and manganese oxides during the oxidative process. Particularly, diffraction peaks of gangue oxides, such as CaO, Al_2_O_3,_ and SiO_2_, are not found in the XRD patterns of the ore mixtures with Mn/Fe mass ratios of 0, 0.1, and 1.2. There are two main reasons for this phenomenon. First, the newborn phase of manganese ferrite has a very strong diffraction peak intensity compared with that of the SiO_2_-bearing phase. Secondly, the few SiO_2_-bearing phases have low crystallinity during the cooling process after high-temperature roasting. Further characterization of the gangue oxides in the roasted pellets was carried out by optical microscopy and SEM-EDS analyses.

### 3.3. Morphology Evolution

**Figure 5:**
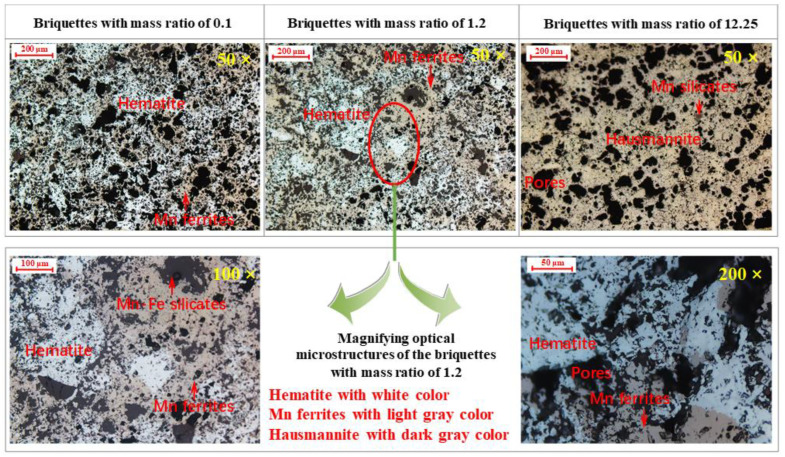
Optical microstructure of the roasted pellets with Mn/Fe mass ratios of 0.1, 1.2, and 12.25.

**Figure 6:**
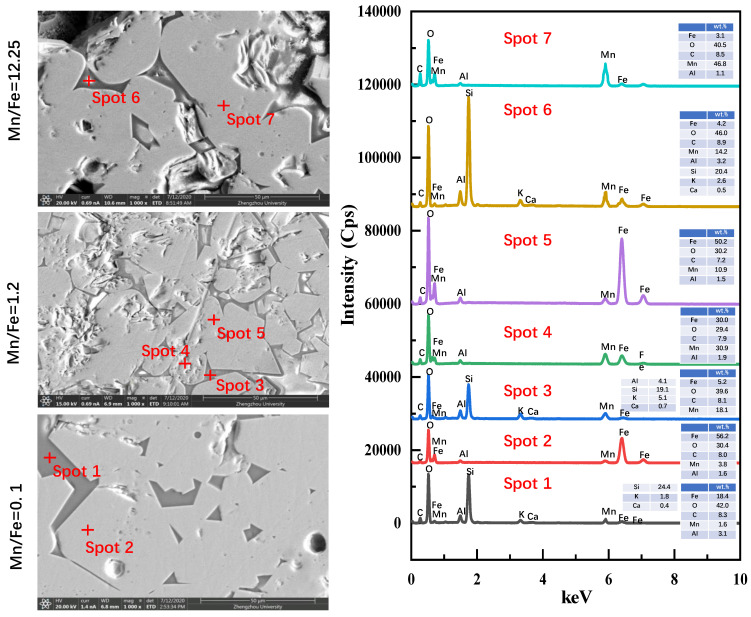
SEM-EDS analyses of the roasted pellets with Mn/Fe mass ratios of 0.1, 1.2, and 12.25.

Based on the characteristics of the CCS and phase results, the pellets with Mn/Fe mass ratios 0.1 (H2), 1.2 (H14), and 12.25 (H19) were selected for morphology investigation. Optical microstructure and SEM-EDS are illustrated in Figure 5 and Figure 6, respectively.

As observed in Figure 5, in the pellets with an Mn/Fe mass ratio of 0.1, Fe_2_O_3_ is recrystallized, and Fe_2_O_3_ particles connect to one another to form bulk grains (white in color). Some manganese ferrites (light gray in color) can be observed around the bulk Fe_2_O_3_ grains. EDS analyses of the pellets with an Mn/Fe mass ratio of 0.1 are shown in Figure 6. Mn content in the Mn ferrites is 3.8 wt.%. This demonstrates that the Mn element can readily diffuse into the crystal lattice of hematite and generate Mn ferrite (Mn_y_Fe_3−y_O_4_). As for the pellets with Mn/Fe mass ratios of 2.0, the area of the hematite phase declines, while the area of the Mn ferrite broadens. As listed in Figure 6, the corresponding Mn contents in the hematite part and the manganese oxide part are 10.9 wt.% and 30.9 wt.%, respectively, indicating increased migration of Mn and Fe elements with increased Mn/Fe mass ratio.

**Figure 7:**
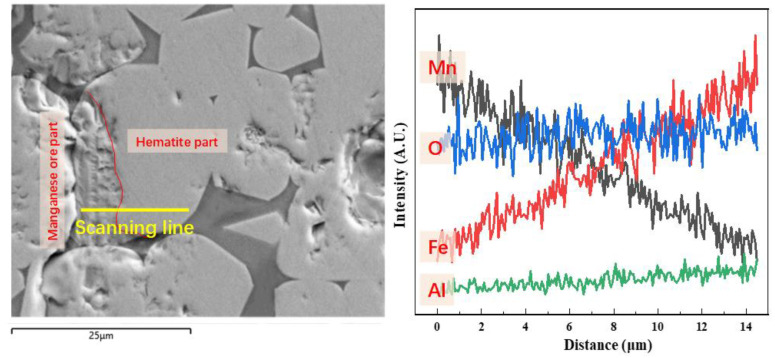
Element line scanning of the roasted pellets with an Mn/Fe mass ratio of 1.2.

Further line-scanning analysis of O, Mn, and Fe elements of the interface layer between the hematite part and the manganese oxide part in the pellets with an Mn/Fe mass ratio of 1.2 is illustrated in Figure 7. Line scanning was conducted from the manganese oxide part to the hematite part. Mn content is higher in the manganese oxide part, and then decreases continuously from the product-layer interface to the hematite part. However, the change rule of Fe content is opposite to that of Mn content. Finally, most formation of Mn ferrites in the pellets of sample H14 is owing to the migration of Fe and Mn elements. With increased Mn concentration in the pellets, the Mn content of sample H14 is higher than that of sample H2.

As to the pure manganese oxide ore pellets (H19), the well-recrystallized hausmannite (Mn_3_O_4_) phase is interconnected to form bulk grains. Note that in all the pellets of samples H1, H14, and H19, some silicates phases (EDS of spots 1, 3, and 6, as shown in Figure 6) are also seen around the borders of the hematite, Mn ferrites, and hausmannite grains. This silicate phase with low melting temperature is also a bonding phase for the consolidation of pellets. However, this liquid-bonding silicate is not the dominant phase, since the volume proportion is less than 10 vol.%. The main consolidation phases in Fe ore, Mn ore, and Fe-Mn ore are recrystallization hematite, hausmannite, and Mn ferrites, respectively.

### 3.4. Discussion of Mineralization

**Figure 8:**
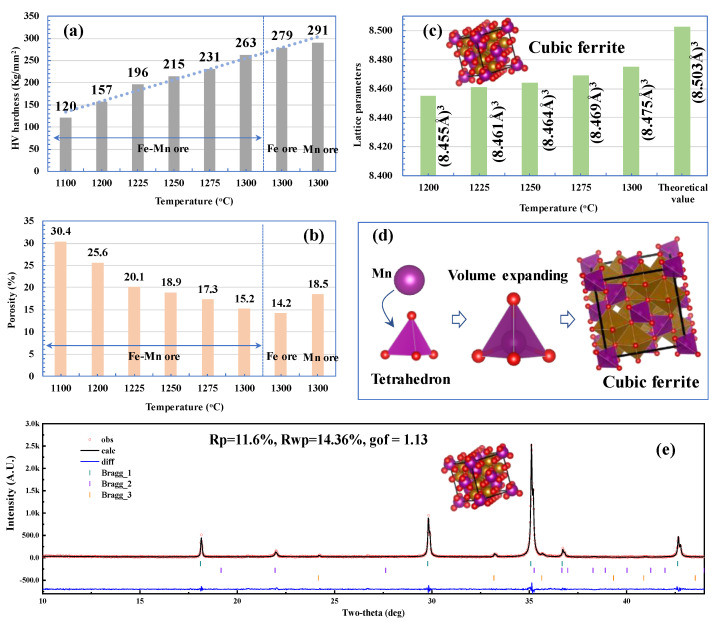
HV hardness, porosity, and lattice parameters of roasted pellets with various Mn/Fe mass ratios. (**a**) HV hardness, (**b**) porosity, (**c**) lattice parameter, (**d**) schematic diagram of element migration, (**e**) XRD-Rietveld refinement.

In order to clarify why the CCS of Fe-Mn ore pellets is lower than that of pure Fe ore and Mn ore, the HV hardness, porosity, and lattice parameters of the Fe-Mn ore with an Mn/Fe mass ratio of 1.2 roasted in an air atmosphere for 10 min are listed in Figure 8. During the HV hardness test, diamond pyramid heads were pressed on the phases of recrystallization hematite, hausmannite, and Mn ferrites. The HV hardness of the Mn ferrites in the Fe-Mn ore increases from 120 kg/mm^2^ to 263 kg/mm^2^ as the roasting temperature increases from 1100 °C to 1300 °C. However, the HV hardness of the Fe ore and Mn ore pellets is higher than that of the Fe-Mn ore pellets. This is consistent with the CCS test results shown in Figure 3. As shown in Figure 8b, the porosities of the Fe-Mn ore pellets decrease from 30.4 vol% to 15.2 vol% with a temperature increase from 1100 °C to 1300 °C. This indicates the crystallization of Mn ferrites is the result of enhanced element migration at elevated temperatures. The effect of temperature on the lattice parameters of the cubic ferrite phase, as calculated by XRD-Rietveld refinement, is shown in Figure 8c. As observed, the lattice parameters increase from 8.455 Å to 8.475 Å with a temperature increase from 1100 °C to 1300 °C. The increasing lattice parameters are ascribed to more Mn ions entering the tetrahedron interstice of the spinel-type structure of ferrites, according to the schematic diagram in Figure 8d. It is well known that lattice parameters increase as doped atoms enter into the interstice of the original structure cells. On the other hand, elevated temperature has a favorable influence on the migration of Mn ions. Therefore, the lattice parameters of the ferrite phase in Fe-Mn ore increase with an increase in roasting temperature. In general, poorly crystallized Mn ferrites with apparent Mn and Fe element concentration gradients result in poor mechanical strength of Fe-Mn ore pellets.

As for the iron ore pellets, the main iron-bearing phase also exists in the form of Fe_2_O_3_, since the theoretical decomposition temperature of Fe_2_O_3_ to Fe_3_O_4_ (Fe_2_O_3_ = Fe_3_O_4_ + O_2_) is 1389 °C in an air atmosphere [29,30]. However, after roasting at 1200–1300 °C, the main consolidation phase in the iron ore pellets transforms from tiny Fe_2_O_3_ particles to bulk structure due to the recrystallization of Fe_2_O_3_. On the other hand, with regard to the oxidization roasting of manganese ore pellets, Mn_3_O_4_ is readily generated from the serial decomposition of MnO_2_ according to reactions (1) and (2). The theoretical decomposition temperatures for the formation of Mn_2_O_3_ and Mn_3_O_4_ are 512 °C and 973 °C, respectively. This indicates that MnO_2_ first decomposes to Mn_2_O_3_ and then further decomposes to Mn_3_O_4_. Therefore, the main consolidation phase in pure manganese ore pellets is well-crystallized hausmannite (Mn_3_O_4_). In brief, pure hematite and manganese ore pellets have well-crystallized hematite and hausmannite (Mn_3_O_4_), respectively, which provide preferable mineralogy for the consolidation of the roasted pellets.

During the roasting of Fe-Mn ore, interactions between manganese oxides and iron oxides occur simultaneously during the decomposition process of MnO_2_. In fact, Mn ferrites are generated from decomposed Mn_2_O_3_, Mn_3_O_4_, and Fe_2_O_3_ according to reactions (3) and (4). However, in roasted Fe-Mn ore pellets, the main consolidation phase is bulk particles, which are composed of hematite and Mn ferrites. However, the newborn Mn ferrite phase has a poorly crystallized boundary, and the ferrite grains have a distinct Mn and Fe element concentration gradient. In other words, the elements are not evenly distributed in the consolidation phase. This gives rise to a lower CCS value for Fe-Mn ore pellets. On the other hand, XRD-Rietveld refinement demonstrated that the increasing lattice parameters of Mn ferrites can be ascribed to more Mn ions entering the tetrahedron interstice of the spinel-type structure of ferrites with increasing roasting temperature, which can be described according to reaction (5). It is inferred that the poorly crystallized Mn ferrites are the result of inadequate diffusion of the Mn ions. In addition, some Mn silicates are also found in the sample H19, and the silicates are derived from the reaction of Mn_3_O_4_ and SiO_2_, based on the reactions (6) [29]. Based on the aforementioned analyses, the corresponding phase and microstructure results, as well as possible reactions, are summarized in Table 2.

4MnO_2_ → 2Mn_2_O_3_ + O_2_, ∆G^θ^ = 151638.77−193.15T (J/mol) (T > 512 °C) (1)

6Mn_2_O_3_ → 4Mn_3_O_4_ + O_2_, ∆G^θ^ = 205028.55−164.56T (J/mol) (T > 973 °C) (2)

2Mn_2_O_3_ + 4Fe_2_O_3_ → 4MnFe_2_O_4_ + O_2_, ∆G^θ^ = 275435.92−189.67T (J/mol) (T > 1179 °C) (3)

2Mn_3_O_4_ + 6Fe_2_O_3_ → 6MnFe_2_O_4_ + O_2_, ∆G^θ^ = 337677.14−242.59T (J/mol) (T > 1118 °C) (4)

[Fe^3+^][Fe^2+^Fe^3+^]O_4_ + [Mn^3+^][Mn^2+^Mn^3+^]O_4_ → [Me^2+^_x_Me^3+^_l−x_][Me^2+^_1−x_Me^3+^_1+x_]O_4_, (Me refers to Mn and Fe) (5)

Mn_3_O_4_ + SiO_2_ → MnSiO_3_ + Mn_2_O_3_ (6)

### 3.5. Suggestions for Consolidation of Fe-Mn Ore Pellets

One reason for the poor mechanical strength of Fe-Mn ore pellets is the formation of poorly crystallized Mn ferrites (Mn_y_Fe_3−y_O_4_). Fortunately, the formation of Mn_y_Fe_3−y_O_4_ is closely related to roasting temperature and time. The migration of elements and formation degree of Mn_y_Fe_3−y_O_4_ increase with increased roasting temperature and time [31,32]. Therefore, in order to ensure good crystallization of Mn ferrite grains, it is suggested to increase the oxidative roasting temperature and time in order to enhance the migration of Fe and Mn elements.

**Figure 9:**
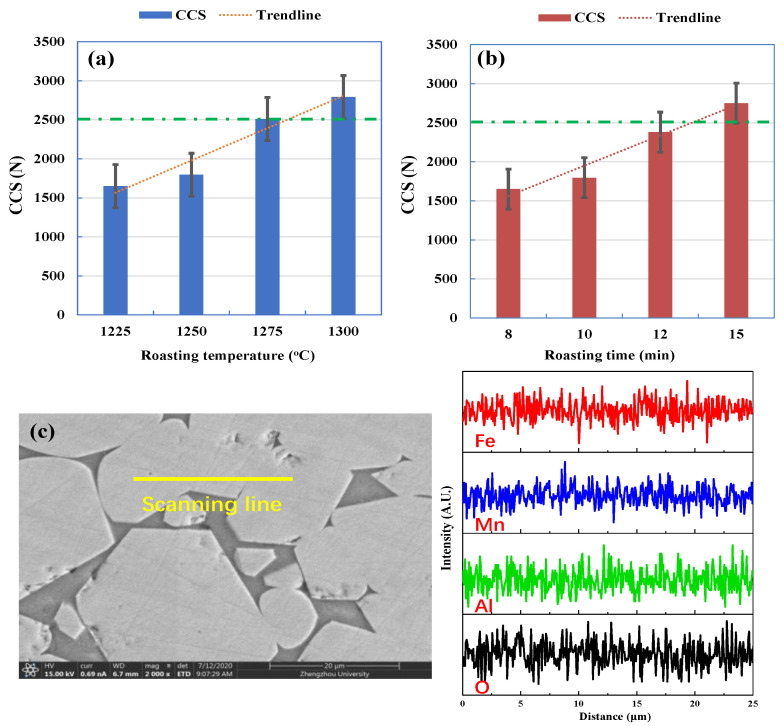
Effect of roasting temperature and time on CCS and element distributions of pellets with an Mn/Fe mass ratio of 1.2. (**a**) Effect of temperature, 10 min; (**b**) effect of time, 1250 °C; (**c**) element distributions of pellets roasted at 1300 °C for 10 min.

In an attempt to improve the CCS of Fe-Mn ore pellets, the effect of roasting temperature and time on CCS and element distributions of pellets with an Mn/Fe mass ratio of 1.2 is displayed in Figure 9. It can be seen that with increased temperature from 1250 °C to over 1275 °C and extended roasting time from 10 min to 15 min, the CCS of Fe-Mn ore pellets is enhanced from 1795 N to 2510 N and to 2750 N, respectively. Mn, Fe, O, and Al elements are uniformly distributed in the well-crystallized Mn ferrite grains, which have an average size of over 20 μm (as seen in Figure 9c). The obtained Fe-Mn ore pellets are superior burden and can be used an acceptable feed (CCS over 2500 N) for Mn alloy production. Finally, a higher temperature of 1275 °C or a longer roasting time of 15 min is recommended for the oxidative consolidation of Fe-Mn ores.

## 4. Conclusions

The oxidative consolidation behavior of manganese ores with various Mn/Fe mass ratios was investigated in this work to determine the cold compression strength (CCS), phase transformation, morphology evolution, and element distribution of pellets. The results can be summarized as follows:

(1) It was found that cold compression strength (CCS) of the pellets first decreases and then increases with increasing Mn/Fe mass ratio from 0 to 12.25 but with a nadir at an Mn/Fe mass ratio of 1.2. The CCS of Fe-Mn ore pellets was observably lower than that of pure hematite and manganese ore pellets, indicating that Mn/Fe mass ratio has an unfavorable effect on the consolidation of Fe-Mn ores. In particular, pellets with an Mn/Fe mass ratio in the range of 0.40–2.0 have a CCS of less than 2500 N, and these pellets cannot satisfy the strength criteria of the pellets required for a large blast furnace.

(2) Phase and morphology results showed that the primary consolidation phases in pure hematite and manganese oxide ores are well-crystallized hematite and hausmannite (Mn_3_O_4_), respectively. However, in Fe-Mn ore pellets, the crystallographic particles contain both hematite and Mn ferrite phases, and the newborn Mn ferrites have evident Mn and Fe element concentration gradients as a result of inadequately diffused metal ions under lower temperatures and short induration time. This results in the poor mechanical properties of Fe-Mn ore pellets. However, the HV hardness and crystallinity of Mn ferrites in Fe-Mn ores increase with increased temperature from 1100 °C to 1300 °C.

(3) In order to obtain Fe-Mn ore pellets with good mechanical properties, a higher temperature (1275 °C) or a longer roasting time (15 min) is required for the oxidative consolidation of Fe-Mn ores. In such optimized cases, Mn, Fe, O, and Al elements are uniformly distributed in well-crystallized Mn ferrite grains, which provide favorable mineralogy for the consolidation of Fe-Mn ores.

## Figures and Tables

**Table 1:** Batching scheme and chemical compositions of the hematite and manganese ore mixtures.

Test No.	Proportion (wt.%)		Mn/Fe Mass Ratio	Chemical Compositions of the Mixtures (wt.%)						
	Hematite Ore	Manganese Ore		TFe	TMn	CaO	MgO	Al_2_O_3_	SiO_2_	Mn + Fe
H0	100	0	0	64.76	-	0.14	0.08	1.7	4.65	64.76
H1	93.6	6.4	0.05	60.89	3.04	0.15	0.09	1.87	4.86	63.93
H2	88.0	12.0	0.1	57.44	5.75	0.15	0.09	2.02	5.04	63.19
H3	82.9	17.1	0.15	54.37	8.16	0.16	0.10	2.15	5.21	62.53
H4	78.4	21.6	0.2	51.61	10.32	0.17	0.10	2.27	5.36	61.94
H5	74.3	25.7	0.25	49.12	12.28	0.17	0.11	2.38	5.49	61.40
H6	70.6	29.4	0.3	46.86	14.06	0.18	0.11	2.48	5.61	60.92
H7	64.1	35.9	0.4	42.90	17.17	0.18	0.12	2.65	5.83	60.07
H8	53.9	46.1	0.6	36.71	22.03	0.20	0.13	2.92	6.16	58.74
H9	46.3	53.7	0.8	32.07	25.68	0.20	0.13	3.12	6.41	57.74
H10	40.4	59.6	1.0	28.49	28.49	0.21	0.14	3.28	6.60	56.98
H11	35.7	64.3	1.2	25.61	30.74	0.22	0.14	3.40	6.76	56.36
H12	30.2	69.9	1.5	22.25	33.39	0.22	0.15	3.55	6.94	55.64
H13	25.9	74.1	1.8	19.67	35.42	0.23	0.15	3.66	7.08	55.08
H14	23.6	76.4	2.0	18.26	36.52	0.23	0.16	3.72	7.16	54.78
H15	19.0	81.0	2.5	15.48	38.70	0.24	0.16	3.85	7.31	54.19
H16	15.7	84.3	3.0	13.44	40.31	0.24	0.16	3.93	7.42	53.72
H17	13.1	86.9	3.5	11.87	41.54	0.24	0.17	4.00	7.50	53.41
H18	8.0	92.0	5.0	8.79	43.96	0.25	0.17	4.14	7.67	52.75
H19	0	100	12.25	3.90	47.80	0.26	0.18	4.35	7.93	51.70

**Table 2:** Consolidation characteristics of the hematite and manganese ore mixtures after roasting.

Ore Types	Main Consolidation Phase	Main Reactions	Microstructure
Hematite ore	Hematite	Recrystallization of Fe_2_O_3_	Fe_2_O_3_ particles connect to form bulk grains [19].
Hematite and manganese ore mixture	Hematite, manganese ferrites	Recrystallization of Fe_2_O_3_ Equations (1)–(5)	Bulk particles contain both hematite and Mn ferrite phases, but the poorly crystallized Mn ferrites have apparent Mn and Fe element concentration gradients due to inadequate diffusion.
Manganese ore	Hausmannite, manganese silicates	Equations (1), (2) and (6)	Pyrolusite decomposes to hausmannite, many pores are formed, and hausmannite particles connect to form bulk grains.

## Data Availability

The data presented in this study are available on request from the corresponding author.
